# Case Report: Secondary Hemophagocytic Lymphohistiocytosis With Disseminated Infection in Chronic Granulomatous Disease—A Serious Cause of Mortality

**DOI:** 10.3389/fimmu.2020.581475

**Published:** 2020-12-09

**Authors:** Jacqueline D. Squire, Stephanie N. Vazquez, Angela Chan, Michele E. Smith, Deepak Chellapandian, Laura Vose, Beatriz Teppa, I. Celine Hanson, Ivan K. Chinn, Lisa Forbes-Satter, Filiz O. Seeborg, Sarah K. Nicholas, Caridad A. Martinez, Carl E. Allen, Thomas J. Connors, Prakash Satwani, Maria Shtessel, Hanadys Ale, Lenora M. Noroski, Nicholas L. Rider, Joshua D. Milner, Jennifer W. Leiding

**Affiliations:** ^1^ Division of Allergy and Immunology, Department of Pediatrics, University of South Florida, St. Petersburg, FL, United States; ^2^ Graduate Medical Education, Memorial Healthcare System, Hollywood, FL, United States; ^3^ Division of Allergy/Immunology and Rheumatology, Department of Pediatrics, Columbia University Irving Medical Center, New York, NY, United States; ^4^ Division of Critical Care Medicine, Department of Pediatrics, Columbia University Irving Medical Center, New York, NY, United States; ^5^ Blood and Marrow Transplant, Johns Hopkins—All Children’s Hospital, St. Petersburg, FL, United States; ^6^ Critical Care Medicine, Johns Hopkins—All Children’s Hospital, St. Petersburg, FL, United States; ^7^ Sections of Immunology Allergy and Retrovirology, Baylor College of Medicine and Texas Children’s Hospital, Houston, TX, United States; ^8^ Division of Pediatric Hematology/Oncology, Texas Children’s Hospital Cancer Center, Houston, TX, United States; ^9^ Division of Hematology/Oncology, Department of Pediatrics, Columbia University Irving Medical Center, New York, NY, United States; ^10^ Division of Allergy and Immunology, Joe DiMaggio Children’s Hospital, Hollywood, FL, United States

**Keywords:** chronic granulomatous disease, hemophagocytic lymphohistiocytosis, sepsis, inflammation, case report, infection, primary immune deficiency

## Abstract

Chronic granulomatous disease (CGD) is a primary immune deficiency due to defects in phagocyte respiratory burst leading to severe and life-threatening infections. Patients with CGD also suffer from disorders of inflammation and immune dysregulation including colitis and granulomatous lung disease, among others. Additionally, patients with CGD may be at increased risk of systemic inflammatory disorders such as hemophagocytic lymphohistiocytosis (HLH). The presentation of HLH often overlaps with symptoms of systemic inflammatory response syndrome (SIRS) or sepsis and therefore can be difficult to identify, especially in patients with a primary immune deficiency in which incidence of infection is increased. Thorough evaluation and empiric treatment for bacterial and fungal infections is necessary as HLH in CGD is almost always secondary to infection. Simultaneous treatment of infection with anti-microbials and inflammation with immunosuppression may be needed to blunt the hyperinflammatory response in secondary HLH. Herein, we present a series of X-linked CGD patients who developed HLH secondary to or with concurrent disseminated CGD-related infection. In two patients, CGD was a known diagnosis prior to development of HLH and in the other two CGD was diagnosed as part of the evaluation for HLH. Concurrent infection and HLH were fatal in three; one case was successfully treated, ultimately receiving hematopoietic stem cell transplantation. The current literature on presentation, diagnosis, and treatment of HLH in CGD is reviewed.

## Introduction

Chronic granulomatous disease (CGD) is a primary immune deficiency caused by defects in one of the five subunits of the NADPH oxidase complex leading to impaired phagocyte respiratory burst and thus increased susceptibility to infections, especially with catalase-positive organisms. In addition to infectious complications, inflammatory and immune dysregulatory complications cause significant morbidity and even mortality (1). Patients with CGD are at increased risk of inflammatory bowel disease, inflammatory respiratory disease, and systemic autoimmune disease (1). They may also be at increased risk of systemic inflammatory disorders, such as hemophagocytic lymphohistiocytosis (HLH) (2, 3).

HLH may present clinically indistinguishable from sepsis or systemic inflammatory response syndrome (SIRS). SIRS criteria are characterized by changes in vital signs (tachycardia, tachypnea, and fever) as well as leukocytosis, leukopenia, or bandemia (4), while HLH features include high fever, cytopenias, and hepatosplenomegaly along with possible coagulopathy, liver dysfunction, elevated triglycerides, and ferritin (5). Lymphocytes and macrophages demonstrating hemophagocytosis may be found on biopsy from the bone marrow, liver, spleen, or lymph nodes. A diagnosis of HLH is established by meeting five of eight clinical criteria (**Table 1**) (5). Though helpful, many of these criteria are not specific to HLH; hemophagocytosis, hyperferrtinemia, hypofibrinogenemia, and thrombocytopenia can occur in sepsis as well (6).

**Table 1:** HLH-2004 diagnostic criteria and patient-specific criteria identified.

HLH criteria:	Patient #1	Patient #2	Patient #3	Patient #4
1. Fever: ≥38.5°C	Yes	Yes	Yes	Yes
2. Splenomegaly	Yes	Yes	Yes	Yes
3. Cytopenia affecting ≥ 2 lineages: - Anemia: Hb <9 g/dl - Thrombocytopenia: platelets <100 k/µl - Neutropenia: ANC <1,000	Yes ✓ ✓ ✓	Yes ✓ ✓	Yes ✓ ✓ ✓	Yes ✓ ✓
4. Hypertriglyceridemia: fasting triglycerides >265 mg/dl Or Hypofibrinogenemia: ≤150 mg/dl	NA Yes	No Yes	Yes Yes	No No
5. Hemophagocytosis in bone marrow, spleen, or lymph nodes	Yes	NA	Yes	No
6. Hyperferrtinemia: >500 µg/L	NA	Yes	Yes	Yes
7. Low or absent NK-cell activity	NA	NA*	NA*	NA
8. Elevated soluble IL-2R/CD25: ≥2,400 U/ml	NA	Yes	Yes	Yes

*✓*cytopenia identified; NA, not available/not attained; *lab drawn, but insufficient sample.

Patients with CGD may present with secondary HLH due to inflammatory disease or infection, the latter being more common (2). Diagnosis of HLH in CGD should prompt physicians and other care providers to actively search for infection, as disseminated infection in CGD can be fatal. Treatment of secondary HLH in CGD should include broad spectrum antimicrobials, including coverage for *Burkholderia*, but may also be complicated by the need for immune suppression to treat hyperinflammation.

## Methods

Charts from four patients with X-linked CGD and who met criteria for HLH were identified and reviewed. Data gathered include genotype, infectious history, clinical course, laboratory parameters, treatment, and where appropriate, cause of death. Patient 3 has been previously published (7), but additional data worthy of submission and relevant to this subject has been included here. Patient 3 is enrolled on institutional protocol allowing for publication and the families of the other patients provided consent for the inclusion of their child’s case in this series.

## Case Reports

### Patient 1

**Figure 1:**
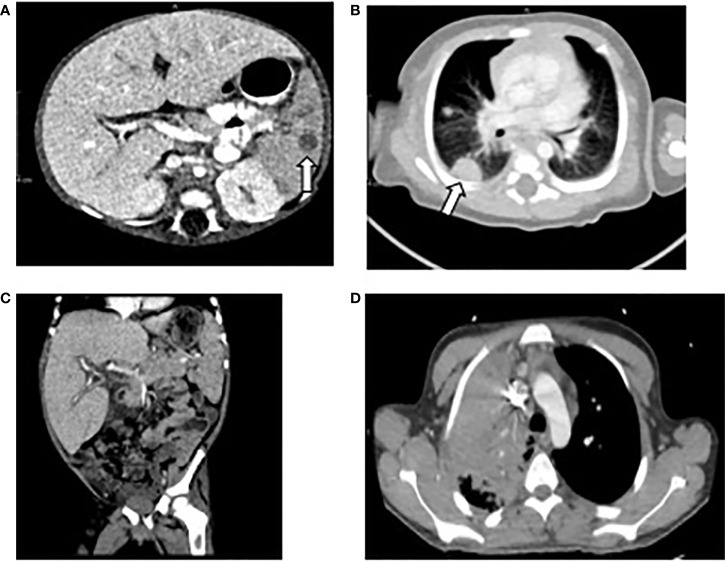
Computed Tomography of chest, abdomen, and pelvis of patients 1, 2, and 4. **(A)** Patient 1: splenic nodule indicated by arrow. **(B)** Patient 1: right sided pulmonary lesion indicated by arrow. **(C)** Patient 2: hepatosplenomegaly. **(D)** Patient 4: right upper lobe pulmonary lesion.

A 32-day-old male with recent diagnosis of X-linked CGD, dihydrorhodamine (DHR) stimulation index (SI) of 1 and known family history (brother with hemizygous mutation in *CYBB* c.742dupA), developed low-grade temperatures (37.8°C). Laboratory evaluation on day 1 of illness was notable for 8% bands without leukocytosis on complete blood count (CBC). Chest x-ray, urinalysis, blood, and urine cultures were not revealing. Low grade temperatures continued, and on day 4 he had temperature to 38.8°C, associated with leukocytosis 16.4 k/mm (3), elevated C-reactive protein (CRP) 11.7 mg/dl, and procalcitonin 0.51 ng/ml. He was hyponatremic 133 mEq/L, hypoalbuminemic 2.7 g/dl, and had elevated transaminases (AST 171 U/L, ALT 164 U/L). Infectious evaluation with blood, urine, and cerebral spinal fluid (CSF) cultures and respiratory pathogen panel by PCR were unrevealing. Empiric treatment was initiated with cefepime and ampicillin. On day 8 of illness, leukocyte count (15.6 k/µl) and CRP (21.5 mg/dl) had further increased and bandemia (15%) was noted. Computed tomography (CT) scan was obtained and demonstrated multifocal pulmonary and splenic nodules measuring up to 13 and 8 mm, respectively (**Figures 1A, B**). Biopsy of the right lung lesion was obtained, and antimicrobials were broadened to meropenem, voriconazole, and micafungin. Fungal etiology was suspected, therefore double anti-fungal coverage was used. Over the next day, bilateral lower extremity mottling and right lower extremity edema with delayed capillary refill were noted. Within hours, the patient progressed to cardiac and respiratory failure requiring mechanical ventilation and multiple vasopressors to maintain adequate perfusion. Hydrocortisone was added as well for vasopressor refractory shock. Repeat laboratory evaluation demonstrated cytopenias, elevated transaminases, and coagulopathy compared to less than 24 h prior (**Table 2**).

**Table 2:** Laboratory evaluation of chronic granulomatous disease (CGD) patients with hemophagocytic lymphohistiocytosis (HLH).

	Patient 1			Patient 2			Patient 3	Patient 4				
	Day 8	Day 9	Day 10	Day 6	Day 7	Day 8	At HLH diagnosis	Day 1	Day 3	Day 30	Day 31	Day 34
**Associated infections**	*Burkholderia cepacia* pneumonia and sepsis			*Penicillium* pneumonia			Disseminated *Candida lusitaniae* ^†^	*Burkholderia multivorans* sepsis				
**WBC** (6–14 k/μl)	10.7	**2.49**	**1.42**	**2.6**	**4.5**	**5.5**	**1.6**	**4.8**	**4.6**	**7.0**	**7.5**	11.5
**Hemoglobin** (10.5–14 g/dl)	**8.7**	**6.9**	7.7*	**7.8**	**10**	**8.7**	**5.9**	**8.7**	**8.8**	10.7	12.2	10.9
**Platelet** (150–450 k/μl)	195	**96**	**18**	**37**	**72**	**53**	**90**	170	162	**64**	**84**	**66**
**ANC** (1,100–6,600)	6,350	**1,050**	**90**					3,570	3,590			
**CRP** (<1 mg/dl)	**25.4**			**11.7**	**13.7**	**15**		**21.2**				
**ALT** (13–50 IU/L)	**141**	**216**	**307**	50	**74**	**123**		**53**		**54**	**65**	**703**
**AST** (22–63 IU/L)	**266**	**656**	**1670**	**245**	**495**	**646**		**86**		**64**	**98**	**3168**
**INR** (0.9–1.1)	**1.32**	**2.77**	**3.73**	**1.5**	**1.4**			**1.5**	**1.4**	**2.4**	**2.6**	
**Fibrinogen** (159–480 mg/dl)	273	**135**	**108^+^**	**116**	**117**	**70**	**80**	529		312	322	272
**Lactate** (0.6–1.1mmol/L)		**6.4**	**>20**							**5.4**	**2.6**	
**Triglyceride** (≤149 mg/dl)				**180**		**196**			**211**		**791**	
**Ferritin** (22–322 μg/L)				**11,531**	**>16,500**	**>16,500**	**11,783**	**2,554**	**2,393**	**34,075**	**65,027**	**>100,000**
**Soluble IL-2R** (<2,400 U/ml)				**23,544**		**18,857**	**17,035**		**16,876**			
**CXCL9** (≤121 pg/ml)					**27,292**					**123.015^¶^**		

*s/p pRBCs, ^+^s/p FFP, cryoprecipitate, **^¶^**collected on day 23, ^†^after treatment for HLH.

**Figure 2:**
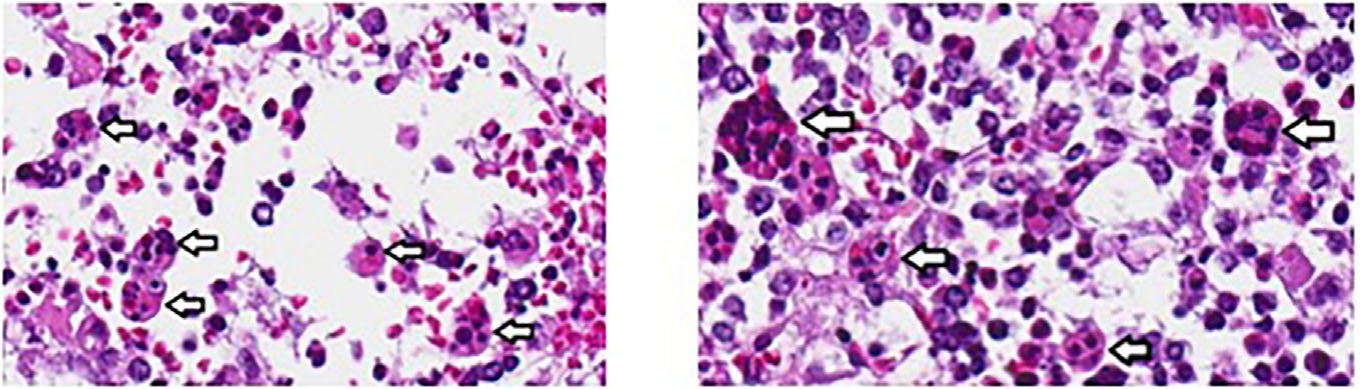
Hemophagocytosis within bone marrow of patient 1, visualized at 40X. Analysis was performed *post-mortem*. Arrows indicate hemophagocytosis.

Over the next 24 h, the patient deteriorated rapidly despite broadening anti-microbials to include vancomycin and amphotericin in addition to meropenem and aggressive resuscitation with multiple blood products. A presumptive diagnosis of HLH was considered based on presence of four of eight known criteria (**Table 1**) and high dose dexamethasone was added to treat his inflammatory response. Despite these efforts, the patient succumbed to multiorgan failure. Blood and tissue biopsy cultures returned post-mortem, yielding *Burkholderia cepacia*. Autopsy confirmed hemophagocytosis within the spleen, liver, and bone marrow (**Figure 2**). Post-mortem next generation sequencing ruled out mutations in genes associated with familial HLH.

### Patient 2

A half Seminole Indian, half African American male was diagnosed with X-linked CGD (DHR SI 1, *CYBB*, c.492delA), after presenting with *Burkholderia gladioli* cervical lymphadenitis at 16 months of age. At 23 months of age, he presented with fevers, diarrhea, and mild cough. Workup revealed a small perianal fistula and mild splenomegaly with tiny subcentimeter T2 hypointense lesions, consistent with granulomas. A biopsy was deemed not possible due to size and location of lesions. Bronchoscopy was normal while endoscopy and sigmoidoscopy showed moderate inflammation of the colon, consistent with CGD-associated colitis. Bronchoalveolar lavage fluid and blood cultures remained without growth at that time. Patient was empirically treated with meropenem, concurrently treated for *Clostridium difficile* infection with oral vancomycin, and corticosteroids 1 mg/kg were initiated for treatment of colitis. Fevers improved and he was discharged home to complete 10 days of antibiotic treatment.

Two days after discharge, he returned with fevers and abdominal distension. He was empirically treated with meropenem and voriconazole. Imaging of the chest and abdomen revealed hepatosplenomegaly with small ascites (**Figure 1C**). He was found to be anemic (hemoglobin 6.9 g/dl) and given blood transfusion. Because of fever, hepatosplenomegaly, and anemia, a diagnosis of HLH was considered. Hyperferritinemia and hypertriglyceridemia were found so methylprednisolone 2mg/kg and high dose intravenous immunoglobulin (IVIG) 2 gm/kg were initiated. Over the next several days, he developed pancytopenia and liver failure with coagulopathy, liver synthetic dysfunction, and elevated liver enzymes (**Table 2**). Laboratory evaluation remained consistent with HLH with high ferritin, CXCL9, and elevated soluble IL-2R (CD25) and no source of infection identified on extensive evaluation. Anakinra was added as adjunct therapy. Three weeks after initial presentation, (1,3)-β-D-glucan assay returned elevated (234 pg/ml) and bronchoalveolar lavage fluid culture from first admission began growing *Penicillium* sp. Amphotericin B was added for additional fungal coverage. On hospital day 10, he developed acute respiratory distress necessitating intubation; imaging was consistent with diffuse pulmonary hemorrhage. Despite aggressive ventilator management, he ultimately progressed to cardiac arrest and resuscitation efforts were not successful. The parents declined autopsy; no further analysis of genes associated with familial HLH was performed.

### Patient 3

An 8-week-old Mexican American boy presented with 2 days of fever (39°C) and 1 week of erythematous rash. The patient was admitted for a sepsis-rule out, empirically placed on ampicillin and cefotaxime following CSF, blood, and urine culture collection. All cultures were negative. His past history was remarkable for contracting pertussis at 10 days of life for which he was treated without complications.

Despite antimicrobial therapy, while still hospitalized, his fevers (38.5–40°C) continued and he developed leukopenia, neutropenia, and anemia. His clinical course further deteriorated with the development of seizures; ceftriaxone and vancomycin were added for empiric meningitis coverage. Hepatosplenomegaly was noted on physical exam and a diagnosis of HLH was considered. Laboratory evaluation showed hyperferrtinemia, hypertriglyceridemia, hypofibrinogenemia, and elevated soluble IL2R level, all consistent with HLH (**Tables 1** and **2**). Perforin expression was normal and NK cell function was inconclusive due to an inadequate sample. A bone marrow biopsy revealed increased histiocyte number with evident hemophagocytosis. HLH treatment was initiated with dexamethasone, cyclosporine, and IVIG. The patient received 8 weeks of therapy and ultimately recovered. However, at week 7 of treatment for HLH, he developed shock and respiratory failure in the setting of abdominal distention with ascites. Blood and peritoneal cultures yielded *Candida lusitaniae*; he was treated with amphotericin B and flucytosine. Additionally, he received eight infusions of granulocytes. Seizures persisted and a brain MRI revealed diffuse infra- and supratentorial leptomeningeal enhancement. Examination of CSF showed pleocytosis, but cultures were negative. He was diagnosed with presumptive fungal meningoencephalitis. Seizures were managed with levetiracetam. An enlarged cervical lymph node was also biopsied which revealed multiple granulomas, many of which had central caseating necrosis and degenerating fungal pseudohyphae.

Given the lymph node histopathology, a DHR assay was performed and noted to be markedly abnormal (SI 1). Next generation sequencing of genes associated with familial HLH was negative; however, assessment for CGD relevant genes revealed a hemizygous mutation in *CYBB* (c.1557delA) and confirmed the diagnosis of X-linked CGD. Profound developmental delays related to his severe illness and fungal meningoencephalitis remained. However, with steady developmental progress he ultimately underwent a matched sibling hematopoietic stem cell transplant (HSCT) following conditioning with busulfan, fludarabine, and alemtuzumab. Now at 7 years of age, he is well and requires no immunosuppression. Seizures are controlled on levetiracetam.

### Patient 4

A 17-year-old previously healthy male presented with several days of fever and increased work of breathing, in the setting of 2 weeks of progressive generalized fatigue and episodic changes in mental status. He was empirically treated with ceftriaxone, vancomycin, and acyclovir for presumed sepsis. Initial laboratory evaluation showed pancytopenia and elevated inflammatory markers. Imaging revealed a large consolidation in the right upper lobe and enlarged lymph nodes (**Figure 1D**). Over the next 2 days, his respiratory distress worsened, and he developed hepatosplenomegaly. He was transitioned to doxycycline and ampicillin-sulbactam for treatment of pneumonia. Given lack of clinical improvement despite broad-spectrum antibiotics, a lung biopsy was performed which revealed necrotizing granulomas. Inflammatory markers continued to rise and a diagnosis of HLH was made with the patient meeting the following criteria: persistent fever, splenomegaly, cytopenia, hypertriglyceridemia, elevated ferritin, and elevated soluble IL-2R levels (**Table 1**); however bone marrow evaluation was normal with no evidence of hemophagocytosis. Because of the patient’s clinical findings and recently revealed family history of X-linked CGD in two nephews, a diagnosis of CGD was suspected and amphotericin was added for fungal coverage. CGD was confirmed with abnormal DHR and next generation sequencing of a panel of genes associated with immunodeficiency identifying pathogenic hemizygous variant c.3G>A in *CYBB*, with no reported variants in primary HLH genes. He was then transitioned to piperacillin-sulbactam and amphotericin, with minocycline for MRSA coverage, followed by initiation of dexamethasone and IVIG, that was met initially with defervescence.

Nineteen days after presentation, he had recurrence of fever and despite treatment with anti-microbials, IVIG, and corticosteroids, he developed profound hypoxemia associated with pulmonary infiltrates and an embolism. Micafungin was added but respiratory failure persisted eventually requiring extracorporeal life support (ECMO) for persistent hypoxemia. On the day of ECMO initiation, his blood culture grew *Burkholderia multivoran* and trimethoprim-sulfamethoxazole was added for additional therapy. Over the next 10 days, inflammatory markers continued to rise, and blood cultures remained persistently positive. Piperacillin-sulbactam was changed to meropenem, levofloxacin was added, and trimethoprim-sulfamethoxazole continued. Because of persistently rising inflammatory markers and marginal clinical improvement, basiliximab, a monoclonal antibody that antagonizes the IL-2 receptor, was administered with drop in ferritin levels from >100,000 to 70,745 ng/ml after administration. A second dose of basiliximab was administered one day later, and ferritin dropped further to 34,075 ng/ml. However, the patient clinically decompensated on this day and developed pulmonary hemorrhage. Basiliximab was administered a third time 6 days later but ferritin levels subsequently rose to 65,027 ng/ml (**Table 2**). Because of the patient’s critical status and uncontrolled inflammation, emapalumab, a humanized monoclonal antibody that binds and neutralizes interferon-gamma (IFN-γ), and granulocyte transfusions were administered as life saving measures. Clinical parameters did not improve, and ferritin remained >100,000 ng/ml after emapalumab. Despite these interventions, the patient developed progressive hypotension and died after failed resuscitation from cardiac arrest, 33 days after initial presentation.

## Discussion

Hemophagocytic lymphohistiocytosis (HLH) is a severe, systemic hyperinflammatory disorder that can either be due to primary or secondary etiologies. Primary HLH refers to genetic disorders associated with HLH and includes a group of autosomal recessive genetic disorders entitled familial hemophagocytic lymphohistiocytosis (FHL) due to mutations in *PRF1* (FHL2), *UNC13D* (FHL3), *STX11* (FHL4), and *STXBP2* (FHL5). In addition, other hereditary disorders associated with primary HLH include Griscelli syndrome type 2 (*RAB27A*), Chediak-Higashi syndrome (*LYST*), and X-linked lymphoproliferative syndromes type 1 and 2 (*SH2D1A* and *XIAP*, respectively) (8, 9). Secondary causes of HLH occur with malignancies, autoimmune disorders, or infections. Besides common bacterial and fungal pathogens, additional infections that have been associated with HLH include human immunodeficiency virus (HIV), herpes viruses, mycobacteria, histoplasmosis, tick-borne bacteria, and in endemic areas, Leishmania (7, 10). The presentation of HLH often overlaps with SIRS or sepsis, making HLH difficult to identify in a critically ill patient. Diagnosis of HLH is established by meeting five of eight criteria based on the HLH-2004 guidelines (5) (**Table 1**) but each of these criteria has been described in sepsis, including elevated soluble IL-2R (CD25) and low or absent NK cell activity (6). While there is not one specific criteria to differentiate HLH from sepsis, ferritin levels >2,000 ng/ml appear to be the most helpful marker to identify HLH (6).

Secondary HLH has been reported to present in a range of primary immunodeficiency disorders, predominantly combined immunodeficiencies such as severe combined immune deficiency, DiGeorge syndrome, and Wiskott-Aldrich syndrome. Systemic Epstein-Barr virus (EBV) and cytomegalovirus (CMV) are the most frequent infectious triggers of secondary HLH reported in combined immunodeficiencies (2). Patients with CGD also can be at high risk for secondary HLH. Bode *et al*. reported a cohort of patients with primary immunodeficiency and HLH, of which one third of patients had previously been diagnosed with CGD (n=22). Of these CGD patients, 86% had an associated infection identified; *Burkholderia cepacia* and *Leishmania* were the most frequent (2). The primary types of bacterial infections associated with secondary HLH are bacteremia and pneumonia; there have been no reported cases of CGD-associated HLH due to viral infection alone (2, 3, 7, 11–13). Secondary HLH, or macrophage activation syndrome (MAS), may also occur due to inflammatory disorders and has been reported in one patient with CGD in association with active uncontrolled colitis, without evidence of concurrent infection (14). MAS is often considered to be on the spectrum of HLH, but is more commonly described in autoimmune conditions such as systemic juvenile idiopathic arthritis and adult onset Still disease (15, 16).

Usually secondary to infection or systemic inflammatory manifestations, HLH can complicate the initial presentation and diagnosis of CGD as seen in patients 3 and 4 in our series (7, 12, 13). Chinn *et al*. reported on the genetic evaluation and diagnosis of primary immunodeficiency in a large cohort of patients with HLH. CGD was among the diagnosis found in this cohort (17). Given that HLH can be a presenting manifestation of CGD and that dire consequences of a missed diagnosis of CGD-associated infection can impact treatment and survival of the patient, evaluation for CGD with a DHR assay should be considered in patients with HLH.

Patients with primary immune deficiencies that develop HLH often present a diagnostic dilemma for clinicians. Due to the high rate of concomitant infection in CGD, it is necessary for a comprehensive search for infectious causes to be sought when a patient presents with possible HLH. This evaluation may include invasive procedures such as bronchoscopy and tissue biopsies as demonstrated in the above cases. Patients with CGD should receive broad-spectrum antibiotic and antifungal coverage upon presentation with targeted therapy once the pathogen and susceptibilities have been determined. Given that *Burkholderia* species are a common cause of sepsis in CGD and frequently associated with HLH in CGD, antibiotics should provide coverage against *Burkholderia (* 2).

HLH-2004 guidelines suggest that initial treatment for HLH include dexamethasone, etoposide, and cyclosporine (5). Due to the risk of further immune suppression in patients with primary immune deficiency diseases, many patients are initially treated with therapies considered more benign, such as IVIG and corticosteroids alone or in combination, which has proven effective in many cases (2, 3, 18). Biologics, including interleukin-1 receptor antagonists (anakinra), anti-interleukin 6 (tocilizumab), and anti-CD52 (alemtuzumab) have been used effectively to treat HLH, but case reports of use in patients with CGD and HLH demonstrate mixed results (7, 19–21). Basiliximab is a chimeric monoclonal antibody directed at the alpha subunit of the IL-2 receptor (CD25) leading to inhibition of T-cell proliferation (22). Use of basiliximab temporarily stabilized inflammatory marker elevation but did not lead to clinical improvement in patient 4. Emapalumab, a humanized monoclonal antibody directed at IFN-γ, recently became the first FDA-approved treatment of pediatric and adult primary HLH with refractory, recurrent, or progressive disease or inability to tolerate HLH therapy (23, 24). Additionally, emapalumab demonstrated dramatic improvement in the treatment of secondary HLH in one patient with severe concurrent viral, bacterial, and fungal infections without known primary immune deficiency (25), but was not effective as a life saving measure in patient 4. Though IFN-γ has been demonstrated to play a critical role in HLH (particularly in murine models), it should be noted that patients with IFN-γ receptor deficiencies also have developed HLH indicating that other cytokines are likely involved in the development of HLH (26). The only potentially curative treatment for primary HLH, as well as CGD, is HSCT. Optimally, control of HLH and any underlying infection should be achieved prior to transplant. Successful subsequent HSCT after HLH or MAS in patients with CGD has been reported (14, 27). Patient 3 in our cohort eventually underwent successful HSCT after HLH was controlled.

In CGD, HLH is almost always secondary to infection. As demonstrated in our four cases, the cause of infection may be difficult to initially identify, leading to delay in targeted antimicrobial treatment and prolonged illness. Based on review of clinical data provided in published case reports, the majority of HLH diagnosed in patients with CGD presented with greater than 1 week of fever and required weeks to months of treatment before resolution of symptoms (3, 7, 10, 12, 13, 18). In addition to lingering symptoms, patients with CGD may develop acute onset of clinical decline, as described in patients 1, 2, and 4 in our cohort (10, 12). Aggressive search and appropriate treatment of infection is paramount in a patient with CGD and fever. Because HLH can overlap with SIRS or sepsis, comprehensive evaluation for signs of HLH should be considered in CGD patients with prolonged fever. Additionally, in patients presenting with HLH and no known immune deficiency, evaluation for CGD should be considered in the diagnostic evaluation. Secondary HLH may complicate disseminated infection in patients with CGD, potentially contributing to morbidity and mortality. Thus, proper treatment of infection is imperative and treatment of hyperinflammation with immune suppression may be necessary for secondary HLH in patients with immune deficiency.

## Data Availability Statement

The original contributions presented in the study are included in the article/supplementary material. Further inquiries can be directed to the corresponding author.

## Ethics Statement

Written informed consent was obtained from the minor(s)’ legal guardian/next of kin for the publication of any potentially identifiable images or data included in this article.

## Author Contributions

JS and JL conceptualized the article. JS, SV, HA, NR, MS, TC, JM, and JL drafted the manuscript. All authors were involved in the patients’ care. All authors contributed to the article and approved the submitted version.

## Conflict of Interest

The authors declare that the research was conducted in the absence of any commercial or financial relationships that could be construed as a potential conflict of interest.
